# Modulation of dendritic cell functions by HTLV-1 with DCs able to direct the presentation of Tax through infected T cells, live virus, and the Tax protein

**DOI:** 10.1186/1742-4690-8-S1-A103

**Published:** 2011-06-06

**Authors:** Sharrón L Manuel, Todd D Schell, Saifur Rahman, Zafar K Khan, Pooja Jain

**Affiliations:** 1 Department of Microbiology and Immunology, and the Drexel Institute for Biotechnology and Virology Research, Drexel University College of Medicine, Doylestown, PA, 18902, USA; 2 Department of Microbiology and Immunology, Pennsylvania State University College of Medicine, Hershey, PA, USA

## 

Human T-cell leukemia virus type 1 (HTLV-1) is the etiologic agent of a debilitating neurologic disorder, HTLV-1-associated myelopathy/tropical spastic paraparesis (HAM/TSP). This disease features a robust immune response including the oligoclonal expansion of CD8+ cytotoxic T lymphocytes (CTLs) specific for the viral oncoprotein Tax. The key pathogenic process resulting in the proliferation of CTLs and the presentation of Tax peptide remains uncharacterized. We have investigated the role of APCs, particularly dendritic cells (DCs), in priming of the anti-Tax CTL response under both in vitro and in vivo conditions. We investigated 2 routes (direct versus indirect) of Tax presentation using live virus, infected primary CD4+/CD25+ T cells, and the CD4+ T-cell line (C8166, an HTLV-1-mutated line that only expresses Tax). Our results indicated that DCs are capable of priming a pronounced Tax-specific CTL response in cell cultures consisting of naïve PBLs as well as in HLA-A*0201 transgenic mice (line HHD II). DCs were able to successfully direct the presentation of Tax through infected T cells, live virus, and cell-free Tax. These observations were comparable to those made with a known stimulant of DC maturation - a combination of CD40L and IFN-γ. Our studies clearly establish a role for this important immune cell component in HTLV-1 immuno/neuropathogenesis and suggest that modulation of DC functions could be an important tool for therapeutic interventions.

